# Pharmacological treatment and current controversies in COPD

**DOI:** 10.12688/f1000research.19811.1

**Published:** 2019-08-29

**Authors:** Mario Cazzola, Paola Rogliani, Daiana Stolz, Maria Gabriella Matera

**Affiliations:** 1 Unit of Respiratory Medicine, Department of Experimental Medicine, University of Rome "Tor Vergata", Rome, Italy; 2 Clinic of Respiratory Medicine and Pulmonary Cell Research, University Hospital of Basel, Basel, Switzerland; 3 Unit of Pharmacology, Department of Experimental Medicine, University of Campania "Luigi Vanvitelli", Naples, Italy

**Keywords:** COPD; treatment; bronchodilators, inhaled corticosteroid

## Abstract

Bronchodilators, corticosteroids, and antibiotics are still key elements for treating chronic obstructive pulmonary disease in the 2019 Global Initiative for Chronic Obstructive Lung Disease (GOLD) recommendations and this is due in part to our current inability to discover new drugs capable of decisively influencing the course of the disease. However, in recent years, information has been produced that, if used correctly, can allow us to improve the use of the available therapies.

## Introduction

In the last 20 years or so, the therapeutic approach to chronic obstructive pulmonary disease (COPD), which the World Health Organization defines as “a lung disease characterized by chronic obstruction of lung airflow that interferes with normal breathing and is not fully reversible” ^1^, has gradually changed with increasing use of a precision medicine approach because we have understood that COPD is a heterogeneous disease ^2^. However, it is becoming increasingly obvious that the current therapeutic approach does not meet all the needs of the patient with COPD; moreover, there is a necessity to identify the patient who best responds to the single class of drugs currently available and also to understand whether new specific combinations of drugs that are more costly and complex are really able to increase the number of patients who can actually take advantage of them ^2^.

In this article, we aim to discuss the evidence and rationale for the Global Initiative for Chronic Obstructive Lung Disease (GOLD) 2019 pharmacological treatment recommendations and the general questions that must to be answered in order to make the best use of bronchodilators in COPD, to define the benefits of combining long-acting bronchodilators versus monotherapy, to explain why triple therapy in COPD is a precision medicine opportunity, and to examine whether and when we can go beyond the GOLD recommendations.

## Bronchodilators as key elements for treating chronic obstructive pulmonary disease

Even in the 2019 GOLD recommendations, bronchodilators are key elements for treating COPD ^3^. The rationale for the use of bronchodilators lies in the mechanisms of bronchomotor tone control and the ability to interfere with them ^4^. There is a direct influence of the parasympathetic nerve (vagus) and an indirect sympathetic influence. This means that if we want to induce bronchodilation, we must block the activation of muscarinic receptors induced by acetylcholine (ACh) with a muscarinic antagonist or we must activate β _2_ adrenoceptors (AR) by using β _2_ agonists ^5^.

The site where a muscarinic antagonist or a β _2_ agonist will act depends mainly on the different distribution of muscarinic receptors and β _2_ ARs in the bronchial tree. There is a decrease in muscarinic receptor density and conversely an increase in the density of β _2_ ARs from central airways to peripheral ones ^6^. However, whereas in the central airways there is a vagal innervation that involves the release of ACh and the consequent stimulation of M _3_ muscarinic receptors present on smooth muscle, in peripheral airways this innervation is absent, but there are M _3 _muscarinic receptors that respond to ACh that is released directly at the epithelium level by the activation of choline acetyltransferase by inflammatory mediators. This is the so-called non-neuronal ACh ^7^. We have explored the influence of aclidinium, a muscarinic antagonist, and formoterol, a β _2_ agonist, on the contractile tone of human segmental bronchi and on the luminal area of human bronchioles and we observed that both drugs are effective in both central and peripheral airways but that aclidinium is more effective in the central airways and formoterol in the peripheral ones ^8^.

However, if we consider the mechanism(s) that can cause an increase in contractility of medium bronchi in the patient with COPD, we soon realize that the intensified parasympathetic activity cannot be opposed by the weak sympathetic tone ^9^. The cross-talk induced by the activation of β _2_ ARs and the inhibition of M _3_ muscarinic receptors at the level of airway smooth muscle cell leads to a synergistic bronchorelaxant effect. Nevertheless, the cholinergic and adrenergic systems certainly influence each other at a post-synaptic and probably at a pre-synaptic level and these complicated interactions explain why combinations of bronchodilators are useful at least from a pharmacological point of view ^10^.

A systematic review with meta-analysis of dual bronchodilation with long-acting muscarinic antagonist/long-acting β _2_ agonist (LAMA/LABA) fixed-dose combinations (FDCs) for the treatment of stable COPD documented that all LAMA/LABA combinations are always more efficient than the respective mono-components in terms of the increase in trough forced expiratory volume in 1 s (FEV _1_) ^11^. LAMA/LABA combinations also improve both transitional dyspnea index and quality of life as expressed by the St. George’s Respiratory Questionnaire scores. There is also evidence that LAMA/LABA FDCs prevent or delay exacerbations of COPD (ECOPDs) when compared with LAMA monotherapy ^12^. Several mechanisms explain why LABA/LAMA may decrease the frequency of ECOPDs ^13^. They can deflate lungs, which results in the stabilization of the airways and thus a decrease in the potential mechanical inflammatory stress; reduce the disproportionate mucus production with an increase in mucociliary clearance; and lower symptom severity. It seems likely that they also cause an anti-inflammatory effect, at least as evidenced *in vitro* and in animal models, but this has not yet been demonstrated in patients with COPD. 

In effect, improvements in FEV _1_, as documented in translational studies, can be explained by a favorable pharmacological synergistic interaction between LAMA and LABA at the level of medium bronchi ^14, 15^. The synergistic interaction also occurs at the level of small airways ^8, 16, 17^, which leads to effective pulmonary desufflation, resulting in decreased dyspnea, enhanced exercise tolerance, and relief of symptoms. Finally, there is evidence that LAMA/LABA combinations inhibit the non-neuronal cholinergic system with a reduction in the release of ACh ^16, 17^, an effect that may explain the protective action against ECOPDs.

Although the 2019 GOLD recommendations suggest using LAMA/LABA only in highly symptomatic patients with a COPD Assessment Test score higher than 20 and a history of two or more moderate exacerbations or one severe exacerbation in the previous year ^3^, we firmly believe that it is useful to start treating COPD with dual bronchodilation from the time of the first diagnosis in order to optimize bronchodilation while interfering with the pathways that influenc airway tone ^18^. At present, we must administer the currently approved doses for treating COPD, but we are confident that the documented pharmacological synergism of action between LAMA and LABA should lead to verifying whether lower dosages can be equally effective. If, as we believe, this approach will be positive, we will certainly succeed in reducing the risk of adverse events that characterize both LAMAs and LABAs when they are taken at the full doses currently approved for the treatment of COPD while satisfying the need to optimize bronchodilation.

## Adding an inhaled corticosteroid

Since there is evidence that inhaled corticosteroid/LABA (ICS/LABA) FDCs decrease ECOPD rates compared with placebo ^19^ and LABA alone ^20^ (although other data do not support this benefit ^21^), great emphasis is given to the use of ICS/LABA combinations in individuals with blood eosinophils of ≥300 cells·µL ^**−1**^ (as this value could allow to appreciate patients with a higher probability of taking advantage from ICS treatment) or in high-risk patients with a history of two or more moderate exacerbations or one severe exacerbation in the previous year at >100 eosinophils·µL ^**−1**^ ^3^. Patients with <100 blood eosinophils·µL ^**−1**^ should not receive ICSs unless they are also asthmatic since this value suggests that these drugs will probably not be able to prevent ECOPDs ^22^. The real problem is represented by the group of patients with 100 to 300 eosinophils·µL ^**−1**^, for whom there is still no solid evidence that allows us to formulate a consistent recommendation; therefore, the decision of whether to add an ICS should be based on individual considerations of probable benefits and possible risks ^22^.

There is a useful pharmacological interaction between corticosteroids and LABAs that may explain why adding ICS to LABA in patients with COPD is convenient ^23^. Corticosteroids increase the numbers of β _2_ ARs, whereas β _2_ agonists induce direct bronchodilation and increase glucocorticoid receptor (GR) nuclear translocation in the presence of corticosteroids, an effect that enhances the anti-inflammatory effects of corticosteroids and also occurs in COPD macrophages that are quite resistant to corticosteroids.

The importance of the ICS/LABA combination also lies in its ability to influence the multicomponent nature of COPD in a much more incisive way than just ICS or LABA alone, thanks to its ability to additively influence the COPD pathophysiology with direct actions on airway obstruction, inflammation, structural changes, and mucociliary dysfunction ^24^.

However, we recently documented that the beclomethasone dipropionate/formoterol furoate combination is able to synergistically relax human bronchi with a consistent effect at low to medium concentrations in small airways, mainly when they have been passively sensitized ^25^. This finding indicates the usefulness of the ICS/LABA combination in the treatment of asthma rather than COPD.

There is evidence that LAMA/LABA FDCs, at least glycopyrronium/indacaterol, prevent or delay ECOPDs when compared with ICS/LABA FDCs ^26^. Unquestionably, the signal generated by the FLAME (Effect of Indacaterol Glycopyrronium Versus Fluticasone Salmeterol on COPD Exacerbations) study, which showed that indacaterol/glycopyrronium was more effective than salmeterol/fluticasone in preventing ECOPDs, is important and suggests the possibility of interrupting the intake of ICS by patients with COPD. However, we still need a cautious approach because we still have doubts regarding COPD patients who can benefit from therapy with ICSs and the fact that double bronchodilation is better than triple therapy (LAMA/LABA/ICS) in the event that the addition of an ICS to the LAMA/LABA combination induces further clinical benefit ^27^.

A practical reason to use ICS-containing triple therapy instead of dual bronchodilation is that triple therapy could reduce all-cause mortality, as demonstrated in the IMPACT (Informing the Pathway of COPD Treatment) trial ^28^, although there are doubts that this actually happens. In fact, the study was not statistically powered for this outcome; in any case, as highlighted by Agusti and colleagues ^22^, this finding may have been influenced by the study’s possible enrollment of patients with a history of asthma. Furthermore, when the number of fatal events induced by the presence of ICS was compared with that observed in the absence of such drugs in a post hoc analysis of the aggregated data of the TRILOGY (Single inhaler triple therapy versus inhaled corticosteroid plus long-acting β _2_ agonist therapy for chronic obstructive pulmonary disease), TRINITY (Single Inhaler Extrafine Triple Therapy versus Long-Acting Muscarinic Antagonist Therapy for Chronic Obstructive Pulmonary Disease), and TRIBUTE (Extrafine Inhaled Triple Therapy versus Dual Bronchodilator Therapy in Chronic Obstructive Pulmonary Disease) trials, the reduction of the risk ratio of fatal events for treatments with ICS compared with treatments without ICS was not statistically significant ^29^.

In any case, stepping-up from dual bronchodilation to triple therapy, an approach that has also been proposed by the 2019 GOLD recommendations ^3^, deliberately ignores the fundamental differences in etiology, severity, and biological substrate of ECOPDs and consequently is not intended to treat the real needs of the patient ^30^. It is important to determine whether and when the addition of an ICS to the LAMA/LABA combination really induces further clinical benefit, regardless of a preventive effect on ECOPDs, and establish the value of this benefit and also determine whether cost differences make the LAMA/LABA combination therapy preferable over triple therapy in real life.

We performed a systematic review and meta-analysis to compare the impact of LAMA/LABA/ICS ** versus LAMA/LABA combination therapy or single long-acting bronchodilator therapy in COPD ^31^. If the outcome was the risk of moderate or severe ECOPDs or the change from baseline in trough FEV _1_, LAMA/LABA/ICS resulted in significantly better outcomes than LAMA/LABA and single long-acting bronchodilator, whereas when the analysis was focused on the risk of pneumonia, no significant differences were observed. However, the person-based number needed to treat (NNT) per year of LAMA/LABA/ICS combination compared with LAMA/LABA combination was significantly lower in patients with ≥300 blood eosinophils·µL ^**−1**^ than in those with <300 eosinophils·µL ^**−1**^. In fact, the NNT to prevent one ECOPD over 1 year was only 8 to 10 patients for those with blood eosinophil counts of at ≥300 cells·µL ^**−1**^ but was about 47 for those with blood eosinophil counts <300 cells·µL ^**−1**^, a number that is likely to be unacceptable for most clinicians ^32^. If pneumonia is considered as the outcome, the person-based number needed to harm (NNH) of LAMA/LABA/ICS combination therapy versus LAMA/LABA combination therapy was 195.34 (95% confidence interval (CI) 85.06–∞) but diminished to 33.89 (95% CI 30.69–37.84) when the only randomized controlled trial that included fluticasone furoate in the triple combination was analyzed. 

The valuation of the effect of triple therapy versus ICS/LABA combination on relevant outcomes in patients with COPD is another critical issue. In a different meta-analysis ^33^, we documented that, compared with ICS/LABA combination, LAMA/LABA/ICS combination significantly improved the trough FEV _1_ from baseline, protected against the risk of moderate or severe ECOPDs, an effect that was not related with the eosinophil level, and did not increase the risk of cardiovascular severe acute events. 

The evidence generated by our meta-analyses on triple therapy suggests that adding an ICS to a LAMA/LABA combination provides modest clinical benefit in the general COPD population, although the effect in the subgroup of patients with eosinophilia is of clinical relevance. Conversely, adding a LAMA to an ICS/LABA combination elicits relevant clinical benefit in the general COPD population, supporting the central role of dual bronchodilation therapy for the treatment of COPD.

Considering the evidence generated by our meta-analyses, we have reached the conviction that in the general COPD population, while the addition of an ICS to a LAMA/LABA combination provides a modest clinical benefit, the addition of a LAMA to an ICS/LABA combination elicits a significant clinical benefit unless the patient presents significant blood eosinophilia. In our opinion, this finding indicates the fundamental role of dual bronchodilation therapy for the treatment of COPD.

The DYNAGITO (Tiotropium and olodaterol in the prevention of chronic obstructive pulmonary disease exacerbations) study, which evaluated tiotropium and olodaterol in the prevention of ECOPDs, documented that the tiotropium/olodaterol FDC did not influence the exacerbation rate compared with tiotropium alone ^34^. In our opinion, a major issue biased the study. The majority of patients (70%) were taking an ICS at baseline ^35^. Patients taking an ICS at baseline did not stop this treatment. This means that a huge number of individuals in the LAMA/LABA group received triple combination therapy (tiotropium/olodaterol plus ICS) whereas those in the tiotropium group actually received tiotropium plus ICS. We believe that the results of this study suggest that tiotropium combined with an ICS is almost as effective as the tiotropium/olodaterol plus ICS combination in lowering the ECOPD risk. Therefore, an ICS/LAMA combination might be considered an option for treating COPD in patients with a history of ECOPDs.

However, we investigated the pharmacological interaction between beclomethasone dipropionate and glycopyrronium on human airway smooth muscle tone and showed that the combination of these two drugs synergistically increased the relaxation of passively sensitized medium and small bronchi, an effect that was not found in non-sensitized bronchi ^36^. The synergistic interaction effect was related to a rise in cAMP concentrations. These findings suggest that ICS/LAMA should be considered a useful therapy for most patients with asthma rather than patients with COPD.

## Further therapeutic options

The 2019 GOLD recommendations suggest adding roflumilast when the response to LAMA/LABA/ICS therapy is not satisfactory in patients with a FEV _1_ of <50% and chronic bronchitis ^3^. In effect, the REACT (Roflumilast in the Prevention of COPD Exacerbations While Taking Appropriate Combination Treatment) study documented that roflumilast causes a reduction in ECOPDs and hospitalizations in patients with severe COPD and chronic bronchitis who had at least two ECOPDs in the previous year despite triple therapy ^37^. At present, roflumilast is the only phosphodiesterase 4 (PDE _4_) inhibitor that has received marketing approval as a maintenance treatment in patients with COPD. However, it increases the risk of adverse events, mainly gastrointestinal side effects, but also induces headache, back pain, and insomnia ^38^. Therefore, it is always crucial to identify not only the right clinical COPD phenotype but also the right type of biological ECOPDs in the hope that, in this way, the use of roflumilast will induce benefits that far outweigh the possible risks ^39^. However, to create further confusion, there is the recent documentation that roflumilast was unable to impact the number of CD8 cells in bronchial submucosa compared with placebo after 16-week treatment but it reduced the eosinophil count in the airways, which suggests the hypothesis that the benefits of roflumilast in COPD could be due to an effect on lung eosinophils ^40^.

Also, theophylline is a PDE inhibitor but is non-selective. At the conventional doses, it is a weak bronchodilator but it is impossible to increase the dose owing to the risk of adverse effects that can also be severe ^41^. Nonetheless, at low concentrations, theophylline causes anti-inflammatory effects in COPD and also enhances histone deacetylase 2 activity, reversing corticosteroid resistance in COPD and further reducing inflammation ^41^. However, there is evidence generated by a meta-analysis of seven observational studies that theophylline increases, albeit marginally, deaths from all causes in patients with COPD ^42^. This information should not be overlooked when prescribing theophylline, at least in some patients with COPD.

Doxofylline differs from theophylline in the pharmacological profile and for this reason should not be considered a modified theophylline ^43^. Apparently, there are no substantial differences in regard to the changes in FEV _1_ from baseline between doxofylline, aminophylline, and theophylline, which are all more effective than bamiphylline ^44^. In contrast, doxofylline, similarly to bamiphylline, appears to be much safer than aminophylline and theophylline. Consequently, we support the use of doxofylline, especially in those patients with COPD who have difficulties in using inhalers or who are not adequately controlled from other pharmacological classes because it is a safe, effective, and relatively inexpensive drug.

The 2019 GOLD recommendations suggest adding azithromycin as an alternative to roflumilast in former smokers ^3^. There is evidence that macrolides reduce inflammation and cellular damage in the inflamed airway with different actions not related to antimicrobial activity that seem to be polymodal and mainly due to inhibition of the transcription factors regulating the inflammatory pathways ^45^. Chronic treatment with erythromycin or azithromycin could effectively reduce the ECOPD rate but may cause increased adverse events and an increase in macrolide-resistant bacteria ^46^. We are definitely against such use of antibiotics, mainly because the approach is too general and does not consider the uniqueness of the subject receiving treatment and therefore is unsuitable for satisfying the individual needs of the patient ^47^. In fact, microanatomical geographic variations in bacterial composition, dependent on the location of the tissue sampling, may exist within the same diseased lung, and the composition of the lung microbiome is influenced by smoking, severity of COPD, ECOPDs, and use of steroids or antibiotics (or both). Furthermore, increasing evidence supports the role of changes in the composition of the microbial community outside of the lung (for example, intestinal and skin microbiota) in modulating chronic lung disorders and respiratory infections.

GOLD also recommends using mucolytic and anti-oxidant agents (N-acetylcysteine, carbocysteine, and erdosteine) because a long-term treatment with these drugs may reduce ECOPDs and cause a small improvement in health status in patients who are not receiving ICS therapy ^3^. This rank of effectiveness was provided by a recent network meta-analysis: erdosteine > carbocysteine > N-acetylcysteine ^48^. Only erdosteine reduced the risk of experiencing at least one ECOPD and the risk of hospitalization due to ECOPDs. Erdosteine and N-acetylcysteine significantly reduced the duration of ECOPDs.

These agents, which are thiol-containing drugs, act also as anti-oxidant and anti-inflammatory agents, are able to modulate human bronchial tone, and elicit anti-infective activity linked to their ability to reduce bacterial adhesion to surfaces and disrupt mature biofilms, an effect that improves the efficacy of antibiotic therapy ^49^.

Oxidative stress triggered by inhaled external oxidants or produced from endogenous sources can cause a reduction in natural anti-oxidants ^50^. When oxidants exceed endogenous anti-oxidants, several cellular processes are activated and generate cellular and molecular events that are considered to play a role in the pathogenesis of COPD. There is preclinical evidence that N-acetylcysteine, when tested at high concentrations, is effective in modulating the damaging effect induced by lipopolysaccharide during an ECOPD because it elicits both anti-oxidant and anti-inflammatory effects ^51^.

## Conclusions

We must be grateful to GOLD for helping doctors to understand the importance of COPD and for stimulating the scientific community’s interest in improving the diagnostic/therapeutic approach to this disorder, but we are trapped with the use of bronchodilators, corticosteroids, and antibiotics. It is likely that this is due to the fact that these classes of drug are able to reduce symptoms and often the risk of ECOPDs in many patients. However, it is also true that their use allows the typical “one size fits all” attitude, which makes the choice of treatment for the patient with COPD easier and for this reason it is still preferred by many doctors. On the other hand, we cannot exclude that it is also due to the fact that we are not yet able to identify new effective therapeutics ^52^.

Regrettably, the “one size fits all” approach that considers only symptoms and risk of exacerbations is insufficient to treat COPD. Therefore, we firmly believe that it is correct to go beyond the recommendations GOLD ^2, 53, 54^.

In recent years, considerable efforts have been made to identify new therapeutic approaches. In particular, investigators have tested whether monoclonal antibodies directed against cytokines and chemokines or their receptors could be useful in reducing the inflammatory component of COPD ^55^. Trials with tumor necrosis factor-alpha inhibitors or antagonists of interleukin 1 beta (IL-1β), IL-5, CXCL8 (IL-8), IL-3, or IL-17 have shown little or no therapeutic effect. We believe that this lack of therapeutic effect reflects the complexity of COPD in which there is no dominant cytokine or chemokine. Therefore, it is imperative to test these monoclonal antibodies in well-identified specific endotypes in which some of these cytokines or chemokines could prevail, such as eosinophilic COPD ^55^. In any case, these treatments will be reserved for a very limited number of patients.

A new and more practical approach could be the identification of tractable traits ^56^. For example, it has been suggested that, at least in primary care, the focus of therapy could be on the two major treatable traits in patients with airway disease: eosinophilic airway inflammation and airflow limitation ^57^. The focus should be on the risk of exacerbations as a result of eosinophilic airway inflammation and symptoms due to airflow limitation. However, if we still wish to follow GOLD recommendations, we must improve the use of the available therapies because we still have fundamental questions regarding their use. In any case, in recent years, information has been produced that, if used correctly, can allow us to improve the utilization of the available therapies ^58^.

## Abbreviations

ACh, acetylcholine; AR, adrenoceptor; CI, confidence interval; COPD, chronic obstructive pulmonary disease; ECOPD, exacerbation of chronic obstructive pulmonary disease; FDC, fixed-dose combination; FEV _1_, forced expiratory volume in 1 s; GOLD, Global Initiative for Chronic Obstructive Lung Disease; ICS, inhaled corticosteroid; IL, interleukin; LABA, long-acting β _2_ agonist; LAMA, long-acting muscarinic antagonist; NNT, number needed to treat; PDE, phosphodiesterase
